# Inhibition of PI3K/AKT/mTOR Signalling Pathway Activates Autophagy and Suppresses Peritoneal Fibrosis in the Process of Peritoneal Dialysis

**DOI:** 10.3389/fphys.2022.778479

**Published:** 2022-03-04

**Authors:** Miao Jia, Hong Qiu, Lihua Lin, Shun Zhang, Damei Li, Donghua Jin

**Affiliations:** Department of Nephrology, The People’s Hospital of Suzhou New District, Suzhou, China

**Keywords:** peritoneal dialysis, peritoneal fibrosis, autophagy, PI3K/AKT/mTOR signalling pathway, renal disease

## Abstract

Peritoneal dialysis (PD) is an important part of replacement therapy for kidney failure. However, long-term PD treatment can cause peritoneal fibrosis. Autophagy may be involved in the pathological mechanism of peritoneal fibrosis (PF). Although autophagy is currently known to be involved in course of PF, its specific effects still lack in-depth research. In this experiment, a high-glucose (HG)-induced peritoneal fibrosis rat model was successfully established *via* intraperitoneal injection of HG peritoneal dialysate, and the phosphatidylinositol 3-kinase (PI3K) inhibitor LY294002 and the mechanistic target of rapamycin (mTOR) inhibitor rapamycin were used to treat peritoneal fibrosis rats. In addition, *in vitro* studies of high glucose-induced peritoneal fibrosis were performed using rat peritoneal mesothelial cells (PMCs). *In vivo* and *in vitro* experiments showed that LY294002 and rapamycin effectively inhibited the process of PF induced by high glucose. In addition, LY294002 and rapamycin were found to alleviate fibrosis by eliminating intracellular reactive oxygen species (ROS) levels, promoting the expression of the epithelial mesenchymal transdifferentiation proteins zonula occludens-1 (ZO-1) and E-cadherin, and inhibiting the expression of p-PI3K, PI3K, p-mTOR, mTOR, the fibroblast-specific proteins ferroptosis suppressor protein 1 (FSP1), and alpha-smooth muscle actin (α-SMA). Moreover, LY294002 and rapamycin promoted expression of autophagy-related proteins LC3-II/I, p62, and beclin-1. The current data indicated that inhibition of PI3K/AKT/mTOR signalling pathway activated autophagy and suppressed PF in the process of PD. Therefore, intervention in this signalling pathway may become a research goal for the prevention and treatment of PF, which has important clinical significance.

## Introduction

Currently, the main treatment for chronic kidney disease (CKD) patients developing kidney failure is still renal replacement therapy including haemodialysis and PD. Recent data shows that the total number of PD patients is increasing at an average annual rate of approximately 18% in China (Wu et al., 2016), indicating that PD is gradually becoming one of the main dialysis methods for kidney failure patients. However, the efficiency of PD gradually decreases with the time of PD prolonged and ultrafiltration failure (UFF) finally occurs, resulting in structural remodelling of the peritoneum, and PF (Balzer et al., 2020). It is currently known that epithelial-mesenchymal transition (EMT) is the initial pathological change in PF. Therefore, intervention in the EMT process may find therapeutic targets for the prevention and treatment of PF. Recent studies have found that the PI3K/AKT/mTOR pathway and autophagy are involved in the PF process.

Autophagy is also known as type II programmed cell death. As a highly conserved self-defence mechanism in mammals, autophagy involves the use of lysosomes to degrade intracellular metabolic waste and reuse its degradation products to maintain the homoeostasis of the cell environment (Galluzzi and Green, 2019). Autophagy is regulated by many signalling pathways, among them the PI3K/AKT/mTOR pathway is mostly studied. This pathway is mainly composed of three acting molecules: PI3K, AKT and mTOR (Xu et al., 2020). PI3K is a type of lipid kinase that is abundant throughout the cytoplasm and can catalyse the phosphorylation of phosphatidylinositol at the D3 position (Cantley, 2002; Xu et al., 2020). PI3K is mainly composed of two subunits, the catalytic subunit p110 and the regulatory subunit p85 (Lucas et al., 2016; Xu et al., 2020). Under physiological conditions, the regulatory subunit keeps the catalytic subunit in a low-activity state (Cantley, 2002; Lucas et al., 2016; Xu et al., 2020). When the cell is stimulated by external cytokine signals, the src homology 2 (SH2) domain of the p85 is phosphorylated, which relieves the restriction on the p110, thereby activating PI3K and its downstream signalling pathways (Lucas et al., 2016). AKT, also known as PKB, is a serine/threonine protein kinase. The expressions and abundances of the three subtypes of AKT, AKT1-3, vary in all mammalian cells. When upstream PI3K is activated, phosphoinositide-dependent protein kinase 1 (PDK-1) and mTOR2 mediate the phosphorylation of AKT, which then transferred from the cytoplasm to the cell membrane and becomes p-AKT (Xue et al., 2017). p-AKT is the core of the PI3K/AKT signal transduction pathway. p-AKT transduces signals to mTOR, which activates the ubiquitin-proteasome pathway and regulates autophagy-related genes or other downstream substrates to exert the physiological effect of inhibiting autophagy (Wei et al., 2020). Signal transmission, autophagosome movement, and vesicle fusion in the process of autophagy are all related to the PI3K/AKT/mTOR signalling pathway (Zheng et al., 2019). Therefore, regulating the balance of the PI3K/AKT/mTOR signalling pathway is of great significance to maintaining the homoeostasis of autophagy.

The role of autophagy in PF was confused in a long time as some findings suggested its activation alleviated PF but others indicated opposite functions in PF (Wu et al., 2016, 2018; Yang et al., 2017; Li R. et al., 2019; Shi et al., 2021). Recent study confirmed that autophagy promotes PF and its blockade exerts protecting roles against PF development by suppressing TGF-β/Smad3 signalling pathway both in PMCs and rat models (Shi et al., 2021). Moreover, an increasing number of studies have found that autophagy regulated by the PI3K/AKT/mTOR pathway is closely related to the occurrence and pathological progression of diabetic nephropathy and renal interstitial fibrosis (Li et al., 2016; Lu et al., 2019; Yang et al., 2020), but the specific pathological mechanism by which autophagy is regulated through the PI3K/AKT/mTOR signalling pathway in the occurrence and development of PF in PD patients requires further exploration.

In this study, we established a rat model of PF and a high glucose-induced PF cell model, and verified *in vivo* and *in vitro* that PI3K inhibitor LY294002 and the mTOR inhibitor rapamycin exert anti-peritoneal fibrosis effects through the PI3K/AKT/mTOR pathway during the process of fibrosis promoted by HG conditions. Our findings confirm the protection role of autophagy in PF and inhibition of autophagy through regulating the PI3K/AKT/mTOR pathway may be a promising potential therapeutic strategy in long-term PD patients.

## Materials and Methods

### Rat Peritoneal Mesothelial Cell Culture

The rats were sacrificed with Nembutal (100 mg/kg, intraperitoneally) and the rat PMCs were prepared following the instructions from Zhou and Yu (2016). We isolated rat peritonea under aseptic conditions and dissected and gently ground the peritonea. The tissues were rinsed by sequential passage through 180, 100, and 75 μm sieves at 4°C. The cells were harvested on 75 μm sieves, resuspended in 2 ml of Dulbecco’s modified Eagle’s medium (DMEM), inoculated into 75 cm^2^ culture flasks and incubated at 37°C and 5% CO_2_ for 7–8 days. The majority of cells observed were rat PMCs.

### Quantitative Real-Time PCR

Rat PMC samples were treated with mannitol(100 mM),glucose(100 mM),and glucose (100 mM) plus autophagy activators LY294002(200 μM, S1105, Selleck, China) or rapamycin(200 μM,S1039, Selleck, China) for 24 h, and samples without additional glucose treatment were used as negative controls. Then, the medium was removed from the six-well cell culture plate, and 1× PBS solution (Sangon, Shanghai, China) was added to wash the cells gently. Next, the six-well plate was placed on ice, 800 μl of TRIzol reagent (T9429, Sigma, United States) was added to each sample and repeatedly pipetted to dislodge all adherent cells, and the cells were transferred to 1.5 ml EP tubes. The total RNA of each sample was extracted according to the manufacturer’s instructions and reverse transcribed with the PrimeScript™ RT Reagent Kit (TaKaRa, Japan). qRT-PCR was performed with SYBR Green Detection Mix (TaKaRa, Japan). The relative expression levels of genes in this study were normalised to actin expression, analysed by the 2^–ΔΔ*Ct*^ method, and summarised from separately harvested PMC samples.

### Western Blot Analysis

Peritoneal mesothelial cell samples were treated as described above for qRT-PCR. Total protein was extracted from each sample to prepare cell lysates (Sangon, Shanghai, China), and the lysates were stored at −20°C. The bicinchoninic acid (BCA) protein quantification method was used to ensure that the concentration of each sample was basically equal. Protein samples were subjected to sodium dodecyl sulfate-polyacrylamide gel electrophoresis (SDS-PAGE) and transferred to PVDF membranes with electrophoresis systems (Tanon VE180 and Tanon VE186, Shanghai, China). The PVDF membranes were blocked with 5% (w/v) skimmed milk powder for 2 h and incubated at 4°C overnight with the following primary antibodies p-PI3K (ab278545, Abcam), PI3K (ab154598, Abcam), p-mTOR (ab109268, Abcam), mTOR (ab134903, Abcam), ZO-1 (ab276131, Abcam), E-cadherin (ab181296, Abcam), FSP1 (ab93283, Abcam), α-SMA (ab124964, Abcam), p62 (ab240635, Abcam), LC3 (ab48394, Abcam), and Beclin-1 (ab207612, Abcam). After being washed with 1× PBS solution (Sangon, Shanghai, China) three times, the membranes were incubated with HRP-labelled secondary antibodies (Abcam, United Kingdom). Immunoreactivity was determined with Enhanced Chemiluminescence (ECL) Reagent (Thermo Fisher Scientific, United States). A gel imaging system (Bio-Rad Gel Doc XR+, United States) and software (Bio-Rad Image Lab Software, Version 5.1 and SPSS 20.0) were used for imaging and statistical analysis. GAPDH was used as an internal control to ensure equal protein loading.

### Intracellular Reactive Oxygen Species Generation Analysis

We measured intracellular ROS generation with the fluorescent probe DCFH-DA (S0033M, Beyotime, China), which is hydrolysed and generates non-fluorescent DCFH after passing through the cell membrane. Intracellular ROS oxidise DCFH to produce fluorescent DCF. The level of DCF fluorescence intensity indicates the level of intracellular ROS.

Peritoneal mesothelial cell samples were treated as described above for qRT-PCR or western blotting, washed with cold 1× PBS solution once, resuspended in serum-free DMEM and incubated with DCFH-DA (10 μM) for 30 min at 37°C. The samples were mixed every 3–5 min to ensure good contact between the probes and PMC. After that, the PMC were washed with serum-free DMEM three times and resuspended in 1× PBS solution. Finally, the PMC samples were analysed by flow cytometry (Life Attune NxT, United States). FlowJo 10 software was used for data analysis.

### Determination of 8-Hydroxydeoxyguanosine Levels and Malondialdehyde Content

The 8-OHdG levels and MDA content were evaluated using ELISA kits (spbio, Wuhan, China) according to the manufacturer’s instructions.

### Measurement of Glutathione Peroxidase and Glutathione Activity

Glutathione peroxidase and GSH activity were measured using Glutathione Peroxidase Assay kit (Abcam, United States) and GSH/GSSG Ratio Detection Assay Kit (Abcam, United States) according to the manufacturer’s instructions.

### Confocal Microscopic Analysis

For confocal microscopic analysis, rat PMCs were transfected with enhanced green fluorescent protein (eGFP)-LC3 and red fluorescent protein (RFP)-LC3 expression plasmid using FuGENE HD transfection reagent (Promega, Madison, WI, United States) in cover glass bottom dishes according to the manufacturer’s instructions.

### Establishment of the Rat Peritoneal Dialysis Model

The rat PD model was established following instructions from Li X. et al. (2019). The control group was given 25 mL of sterile normal saline intraperitoneally every day. The PD group was given 4.25% high-glucose peritoneal dialysate intraperitoneally every day, and LPS (0.6 mg/kg) was given intraperitoneally on days 1, 3, 5, and 7 of the experiment to induce chronic inflammation. One week after the start of the experiment, that is, after the intraperitoneal injection of LPS was completed, the PDF+LY294002 group and PDF + rapamycin group were given an intraperitoneal injection of high-glucose peritoneal dialysate and an additional intraperitoneal injection of LY294002 (100 μM/kg) or rapamycin (100 μM/kg) once a day; the time of each administration was basically fixed at approximately 8:00 in the evening. After 4 weeks, the establishment of the peritoneal fibrosis model related to PD was completed. All rats were sacrificed with intraperitoneal injection of Nembutal (100 mg/kg).

### Immunohistochemistry

Paraffin-embedded blocks were cut into 4-μm-thick sections, and the sections were dewaxed and hydrated. Then, the sections were immersed in distilled water containing 3% hydrogen peroxidase twice to reduce endogenous oxidase activity. Next, the tissue sections were incubated with anti-FSP1 antibody (Abcam, ab197896), anti-α-SMA antibody (Abcam, ab124964), anti-ZO-1 antibody (Abcam, ab221546) and anti-E-cadherin antibody (Abcam, ab212059) for 2 h at room temperature, and subsequently, a goat-anti-rabbit antibody was applied to the cells at room temperature for 40 min. The degree of staining was determined by developing with diaminobenzidine (DAB) chromogen (Bio-Rad, Inc., CA, United States). Subsequently, the tissue sections were dehydrated and sealed with gum. Five random fields of view at 100× magnification were selected with a camera using a microscope (Olympus, Japan), and the mean microvessel count was recorded as the microvessel density.

### Statistical Analysis

All data were presented as the means ± SD. Independent group comparisons were analysed using one-way analysis of variance (ANOVA). *P < 0.05 and **P < 0.01 were considered statistically significant and highly significant.

## Results

### LY294002 and Rapamycin Inhibited Reactive Oxygen Species Generation

**FIGURE 1:**
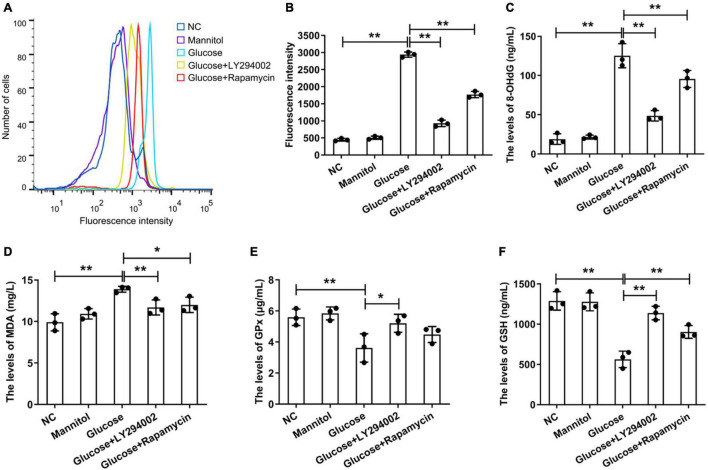
Effects ofLY294002 and rapamycin on HG-induced oxidative stress in rat PMCs. **(A)** Flow cytometry analysis showed that ROS generation in the HG group and the HG + LY294002 or rapamycin group changed compared with that in the NC group. **(B)** Statistical analysis of ROS generation, as determined by flow cytometry analysis. The activities of 8-OHdG **(C)**, MDA **(D)**, GPX **(E)** and GSH **(F)** were analysed by ELISA kits. Columns represent the means (*n* ≥ 3); bars represent the SD; *p < 0.05, **p < 0.01.

Under the stimulation of high glucose and inflammation, a large amount of ROS is produced in rat PMCs, which is the main reason for accelerating cell senescence and apoptosis (Wu et al., 2016). We focussed on the function of LY294002 and rapamycin in the ROS signalling pathways. We performed flow cytometry to analyse the generation of intracellular ROS, and the results showed that the ROS level was significantly increased in the HG group compared with the negative control (NC) group (*P* < 0.01), but it was significantly reduced in the HG + LY294002/rapamycin groups compared with the HG group (*P* < 0.01) (Figures 1A,B). Thus, both LY294002 and rapamycin blocked ROS generation. Additionally, the levels of the DNA oxidative damage marker 8-OHdG in the HG + LY294002/rapamycin groups were significantly decreased compared with the HG group (*P* < 0.01) (Figure 1C). The malondialdehyde (MDA) content was also revealed significantly decreasing in the HG + LY294002/rapamycin groups compared with the HG group (Figure 1D). Moreover, the expression of glutathione peroxidase (GPX) and glutathione (GSH) were both significantly downregulated in HG groups compared to the NC (*P* < 0.01) (Figures 1E,F). LY294002 and rapamycin increased the expressions of GSH significantly in HG + LY294002/rapamycin groups (*P* < 0.01) (Figure 1F). The expression of GPX exhibited similar situations in HG + LY294002/rapamycin groups (Figure 1E). Thus, LY294002 and rapamycin increased the activities of GPX and GSH in HG-induced PMCs.

### Effects of LY294002 and Rapamycin on High-Glucose-Induced Peritoneal Fibrosis *in vitro*

**FIGURE 2:**
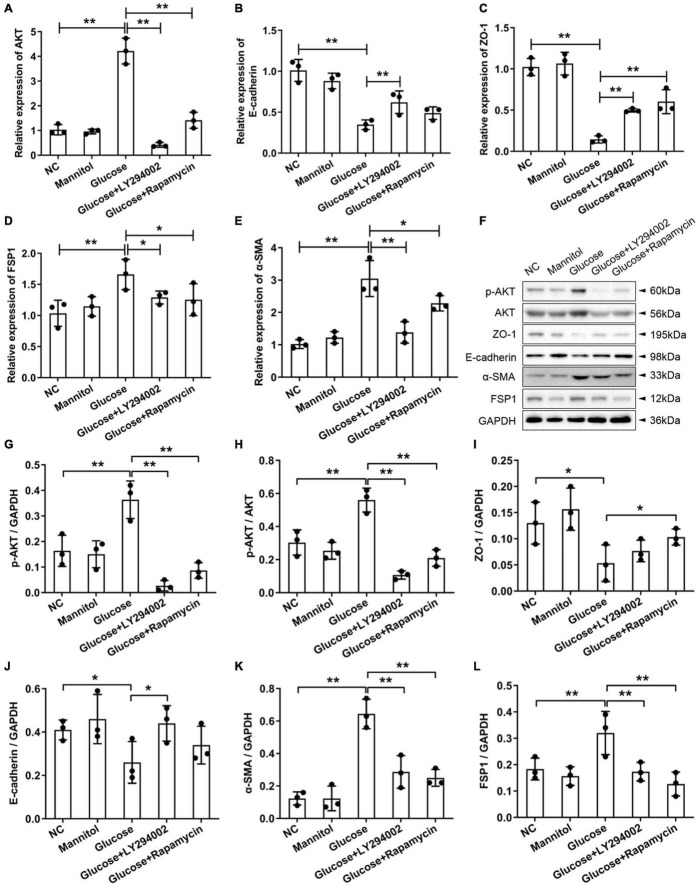
Effects of LY294002 and rapamycin on HG-induced peritoneal fibrosis *in vitro*. **(A–E)** The qRT-PCR results showed the expression of AKT, E-cadherin, ZO-1, FSP1, and α-SMA in the HG group, the HG + LY294002 or rapamycin group, and the NC group. **(F)** The western blot results showed that the protein levels of p-AKT, E-cadherin, ZO-1, FSP1 and α-SMA changed in the HG group and the HG + LY294002 or rapamycin group compared with those in the NC group. **(G–K)** For the quantitative analysis of p-AKT, E-cadherin, ZO-1, FSP1 and α-SMA protein levels, target protein expression was normalised to that of glyceraldehyde 3-phosphate dehydrogenase (GAPDH). **(L)** The quantitative analysis of p-AKT protein expression normalised to AKT. Columns represent the means (*n* ≥ 3); bars represent the SD; *p < 0.05, **p < 0.01.

To examine the effects of LY294002 and rapamycin on the epithelial mesenchymal transdifferentiation markers ZO-1 and E-cadherin and the fibroblast-specific markers FSP1 and α-SMA, we performed qRT-PCR and western blot analysis of rat PMCs. The qRT-PCR results showed that the expression of AKT, FSP1, and α-SMA increased sharply and significantly in the HG groups compared with the NC groups or mannitol groups (*P* < 0.01) (Figures 2A,D,E). The AKT and α-SMA expressions decreased significantly after the addition of LY294002 or rapamycin to the HG solutions (*P* < 0.01, *P* < 0.05) (Figures 2A,E). And the FSP1 expressions were obviously reduced in HG + LY294002/rapamycin groups (Figure 2D). The expression of ZO-1 and E-cadherin revealed opposite results that they were both decreased significantly in the HG group (*P* < 0.01) compared to the NC and increased significantly in the HG + LY294002/rapamycin groups compared to the HG group (*P* < 0.01) (Figures 2B,C). Western blot analysis showed that the FSP1 and α-SMA protein levels increased significantly with HG treatment and decreased obviously after LY294002 or rapamycin supplementation in the HG solutions (*P* < 0.01) (Figures 2F,J,K). Furthermore, ZO-1 and E-cadherin protein levels decreased significantly under HG treatment and increased after LY294002 or rapamycin was added to the HG solution (Figures 2F,H,I). The AKT proteins revealed obvious increasing under HG conditions and decreasing with LY294002 or rapamycin supplementation (Figure 2F). The p-AKT protein expression showed similar variation trend with AKT when normalised to GAPDH or AKT (*P* < 0.01) (Figures 2F,G,L). These confirmed the qRT-PCR results.

### Establishment of High-Glucose-Induced Peritoneal Fibrosis in Rats

**FIGURE 3:**
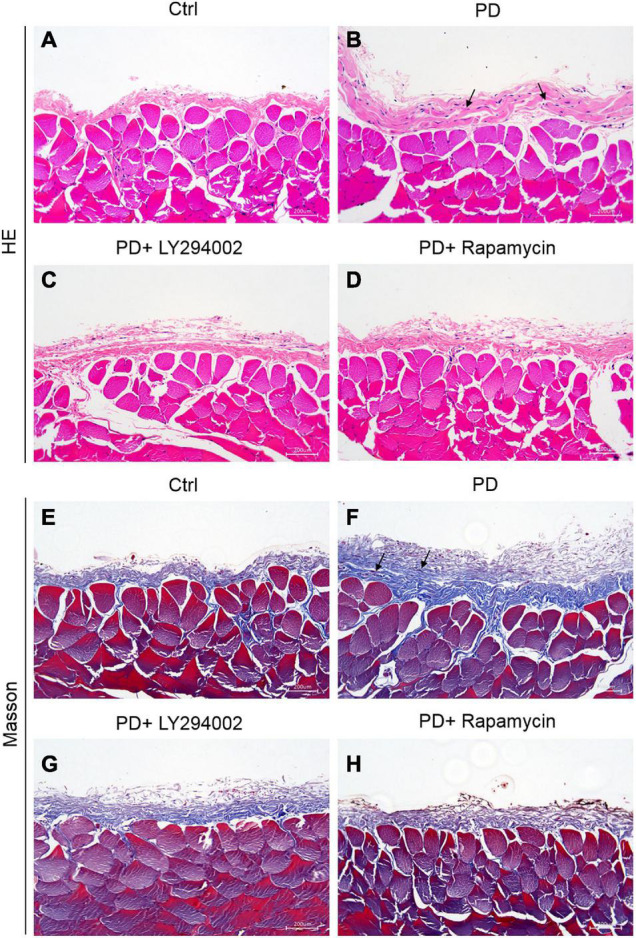
Establishment of HG-induced peritoneal fibrosis in rats. **(A–D)** HE staining was used to analyse the peritoneal tissue in the control group, the HG peritoneal dialysate-induced group and PD rats treated with LY294002 or rapamycin. **(E–H)** Masson staining was used to analyse the peritoneal tissue in the control group, the HG peritoneal dialysate-induced group and PD rats treated with LY294002 or rapamycin. Bar = 200 μm. The location indicated by the black arrow is collagen fibres.

The intervention effect of the high glucose-induced PD model was evaluated by observing the degree of cell infiltration and fibrosis. The results of haematoxylin-eosin (HE) staining showed that the peritoneal tissue of the control group was smooth and thin. The peritoneum was covered with a thin layer of mesothelial cells connected with connective tissues. There was no obvious accumulation of collagen fibres under the mesothelial cells and no obvious infiltration of inflammatory cells. Compared with that of the control group, the peritoneal tissue of the PD group was significantly thickened, the mesothelial cells fell off, collagen fibres accumulated significantly under the mesothelial cells, and there was obvious inflammatory cell infiltration (Figures 3A,B). Masson staining showed that a small amount of collagen fibres accumulated under the mesothelial tissue of the control group rats, and there were fewer blue collagen fibres, while the peritoneal tissues of rats in the PD group obviously accumulated a large amount of collagen, the coloured collagen fibres were more density, and vascular hyperplasia was obvious (Figures 3E,F). The PD rats treated with LY294002 or rapamycin showed obviously symptom alleviation compared to the PD group (Figures 3C,D,G,H).

### Effects of LY294002 and Rapamycin on High-Glucose-Induced Peritoneal Fibrosis in Rats

**FIGURE 4:**
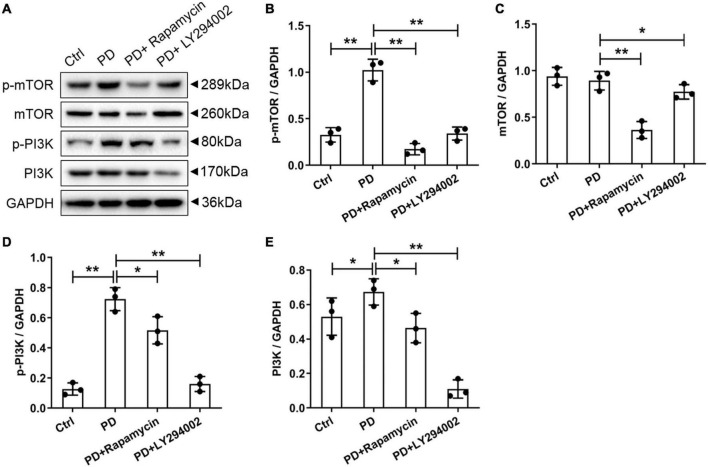
Effects of LY294002 and rapamycin on PI3K/mTOR signalling pathway on HG-induced peritoneal fibrosis in rats. **(A)** The western blot results showed that levels of the p-mTOR, mTOR, p-PI3K and PI3K proteins changed in the PD rats and PD rats treated with LY294002 or rapamycin compared with those in control rats. Three rats in each group were prepared. **(B–E)** For the quantitative analysis of p-mTOR, mTOR, p-PI3K, and PI3K protein levels, target protein expression was normalised to that of glyceraldehyde 3-phosphate dehydrogenase (GAPDH). Columns represent the means (*n* = 3); bars represent the SD; *p < 0.05 and **p < 0.01.

**FIGURE 5:**
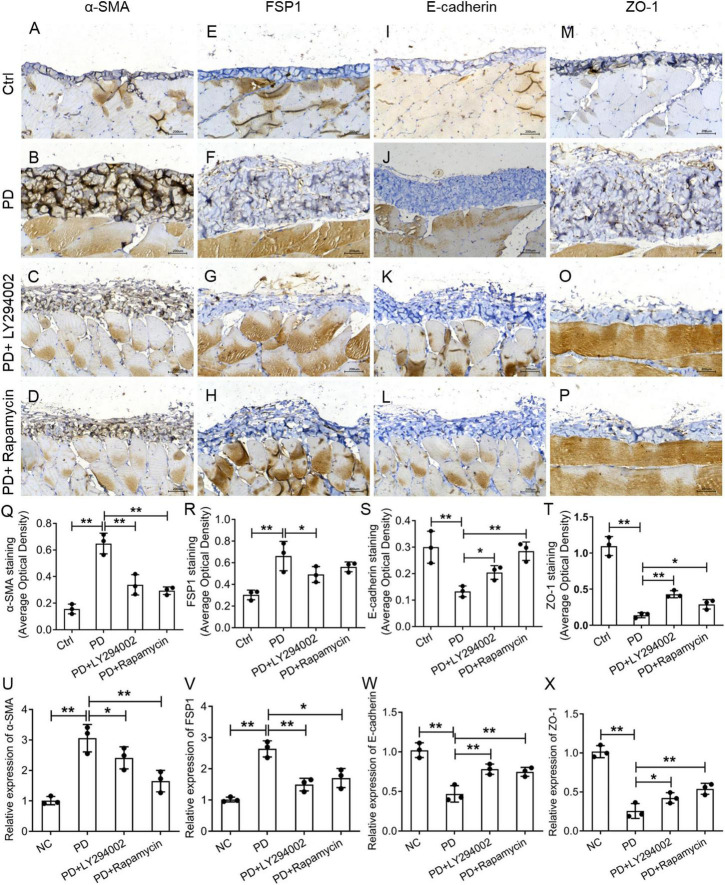
Effects of LY294002 and rapamycin on HG-induced peritoneal fibrosis in rats. The expression of α-SMA **(A–D)**, FSP1 **(E–H)**, E-cadherin **(I–L)** and ZO-1 **(M–P)** in the peritoneum of control rats, PD rats and PD rats treated with LY294002 or rapamycin was determined by IHC assay. Three rats in each group were prepared. **(Q–T)** Quantitative analysis of the average optical density of α-SMA, FSP1, E-cadherin and ZO-1 in the IHC assay. **(U–X)** The qRT-PCR results showed the expression of α-SMA, FSP1, E-cadherin, and ZO-1 in the HG group, the HG + LY294002 or rapamycin group, and the NC group. Columns represent the means (*n* ≥ 3); bars represent the SD; *p < 0.05, **p < 0.01.

To further investigate the relationship between fibrosis and the PI3K/AKT/mTOR signalling pathway in the peritoneum, we performed a western blot analysis of the PD rats to examine the effects of LY294002 and rapamycin on the expression the classical factors in the PI3K/AKT/mTOR signalling pathway: PI3K and mTOR. Western blot analysis showed significantly increased levels of the p-mTOR, mTOR, p-PI3K, and PI3K proteins in PD rats and significantly decreased levels after LY294002 or rapamycin stimulation (Figures 4A–E). Furthermore, immunohistochemistry was performed to assess the expression of ZO-1, E-cadherin, FSP1, and α-SMA in PD rats that were intraperitoneally administered LY294002 or rapamycin. α-SMA-positive cells (Figures 5A–D) and FSP1-positive cells (Figures 5E–H) were mainly observed in PD rats, and α-SMA and FSP1 immunostaining seemed to be increased in the peritoneum of PD rats (*P* < 0.01) and decreased in PD rats treated with LY294002 or rapamycin (*P* < 0.01, *P* < 0.05) (Figures 5Q,R). However, the expressions of E-cadherin (Figures 5I–L) and ZO-1 (Figures 5M–P) were lower in PD rats than in the control rats (*P* < 0.01) and higher in PD rats treated with LY294002 or rapamycin compared with the PD rats (*P* < 0.01, *P* < 0.05) (Figures 5S,T). Taken together, rapamycin and LY294002 inhibited the phosphorylation of PI3K/mTOR proteins to activate autophagy in PD rats. The qRT-PCR results from the PD group and PD rats treated with LY294002 or rapamycin confirmed the immunohistochemistry results in further (Figures 5U–X).

### LY294002 and Rapamycin Promoted Expression of Autophagy-Related Proteins *in vitro* and *in vivo*

**FIGURE 6:**
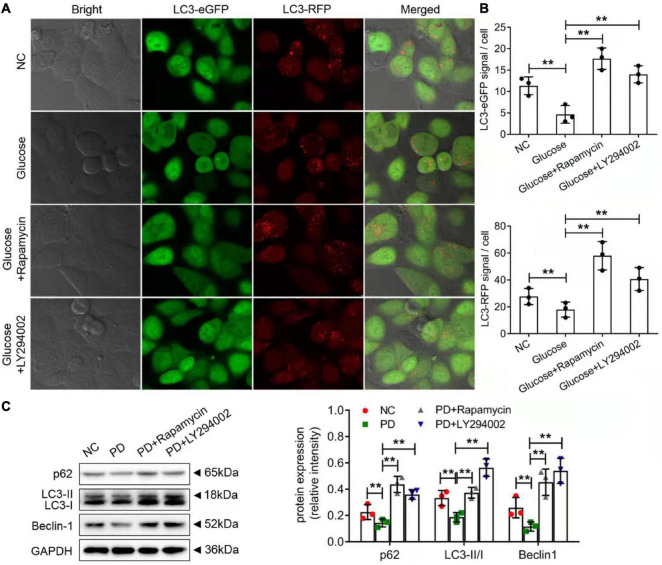
Rapamycin and LY294002 affected autophagy-related degradation processes on HG-induced peritoneal fibrosis *in vitro* and *in vivo*. **(A)** The autophagic cell-death model was replicated *in vitro* using rat PMCs and the autophagy inducer, rapamycin and LY294002. Immunofluorescence staining was performed on the autophagy marker, LC3 protein. **(B)** The quantitative analysis of the result in **(A)**. **(C)** Western blot and quantitative analysis detected autophagy-related protein expression in the peritoneum of control rats, PD rats and PD rats treated with LY294002 or rapamycin. Three rats in each group were prepared. Columns represent the means (*n* ≥ 3); bars represent the SD; **P < 0.01.

The autophagic cell-death model was replicated *in vitro* using rat PMCs and the autophagy inducer, rapamycin and LY294002. Immunofluorescence staining was performed on the autophagy marker, LC3 protein. As compared to NC groups, HG group showed a significantly lower expression of the LC3 protein indicating the onset of autophagy. Upon rapamycin and LY294002 treatment, the LC3 protein was significantly increased reflecting the effect of rapamycin and LY29400 on autophagy induction and its impact on the autophagy-related ubiquitination and degradation processes (Figures 6A,B). Additionally, as compared to control rats, the expression of the autophagy related proteins such as LC3-II/I, p62, and beclin-1 were significantly decreased (*p* < 0.01) in PD rats (Figure 6C). Treatment of rapamycin and LY294002 to PD rats significantly (*P* < 0.01) increased the expression levels of the autophagy facilitating proteins (Figure 6C). The results showed that rapamycin and LY294002 could effectively promote the level of autophagy in the peritoneal fibrosis of rats induced by high glucose.

## Discussion

Current studies have confirmed that EMT accompanied by high expression of PI3K, AKT and mTOR proteins occurs in PMCs under the stimulation of high-glucose dialysate (Deng et al., 2019). A study by Wang et al. (2019) found that there is excessive activation of AKT in peritoneal EMT tissue, suggesting that the PI3K/AKT signalling pathway is involved in the EMT process of peritoneal tissue. After treatment with the PI3K/AKT pathway blocker wortmannin, the expression of p-AKT and α-SMA in PMCs was significantly inhibited (He et al., 2020). These studies show that the PI3K/AKT/mTOR signalling pathway is involved and activated in the process of PF. but the downstream mechanism after activation still lacks further research.

Under stimulation by high glucose and inflammation, PMCs produce a large amount of ROS, which is the main cause of accelerated cell senescence and apoptosis (Wu et al., 2016). ROS destroy PMC mitochondrial membrane integrity and reduce mitochondrial membrane potential (MMP). Moreover, ROS production is accompanied by an increase in intracellular DNA oxidative damage markers such as 8-hydroxydeoxyguanosine (8-OHdG) and a decrease in antioxidants such as glutathione (GSH) (Balzer et al., 2020). Our results showed that in rat PMCs, LY294002 and rapamycin prevented the increase of ROS levels stimulated by high-glucose peritoneal dialysate, increased the activity of GPX and GSH, and reduced the production of 8-OHdG and MDA. These results indicate that the PI3K/AKT/mTOR signalling pathway may be involved in oxidative stress regulating in rat PMCs. As high-glucose-induced ROS generation is considered to be the unifying cause in diabetic complications, inhibition of the PI3K/AKT/mTOR pathway may be benefit to maintain the intracellular redox balance in PD patient treatment (Wang et al., 2018; Lu et al., 2019). Moreover, LY294002 and rapamycin inhibit the expressions of fibroblast-specific proteins FSP1 and α-SMA in rat PMCs stimulated by HG, implying reduced fibrosis. Meanwhile, they upregulated the expressions of epithelial mesenchymal transdifferentiation markers ZO-1 and E-cadherin. These results indicated that in rat PMCs inhibition of PI3K/AKT/mTOR pathway significantly delayed the EMT of PF during dialysis.

The animal results show that the thickness of the peritoneal tissue and the deposition of collagen fibres in the high-glucose peritoneal dialysate group increased significantly. It may be that high glucose in the peritoneal dialysate induced inflammatory hyperplasia and that the corresponding extracellular matrix in the peritoneal tissue was caused by increased secretion. FSP1 and α-SMA were highly expressed in the peritoneal tissue of the PD group, while the expression levels in the PD+LY294002 group and PD+ rapamycin group were lower, which was basically consistent with the results of *in vitro* experiments. This study also found that the expression of the epithelial cell marker molecule E-cadherin was basically the same as that of ZO-1. It was low in the PD group, but increased with the treatment of LY294002 or rapamycin. These results suggested that in an animal model of PF, inhibiting the PI3K/AKT/mTOR signalling pathway can directly interfere with the expression of the fibroblast-specific proteins FSP1 and α-SMA, thereby hindering the occurrence and development of PF which verified the above *in vitro* cell experiments completely. Considering the expression changes of these proteins generally leads to the fibroblastoid appearance in EMT, which is a major initial pathological change of renal fibrosis (Smith and Bhowmick, 2016; Ying and Wu, 2017; Bornes et al., 2019), and under the continuous action of high-glucose-concentration peritoneal dialysate, the intraperitoneal environment may undergo changes such as oxidative stress, increased synthesis of inflammatory factors, which ultimately lead to peritoneal mesothelial EMT in cells (Das et al., 2020), we suggested that inhibiting of the PI3K/AKT/mTOR signalling pathway may suppress the intracellular oxidative stress induced by HG, exerting preventing roles on PF via regulating the expression of epithelial mesenchymal transdifferentiation and fibroblast-specific markers. These findings were consistent with the previous results that ROS plays key roles in EMT progression (Yang et al., 2019; Chatterjee and Chatterjee, 2020).

Autophagy is a self-regulation mechanism used by mammalian cells to resist external pressure or environmental changes (Levine and Kroemer, 2008). And it is considered theoretically that autophagy may be involved in the pathological mechanism of PF. However, the current research results on autophagy and PF seem to be somewhat contradictory. In this study, the expressions of LC3-II/I, p62, and beclin-1, exerting activation roles on autophagy, were significantly increased with the supplement of LY294002 and rapamycin in PD rats. Research by Yang et al. (2017) showed that high-glucose conditions inhibited the autophagy of PMCs and the expressions of Beclin-1, LC3 II-related genes and p62 were decreased. However, they were increased by intervening with 1,25(OH)2D3 or inhibiting the mTOR signallings. Our results confirmed the findings completely. Previous findings suggested that real-time activation of autophagy may inhibit the continuous activation of NLRP3/IL-1β caused by long-term application of high-glucose peritoneal dialysate and delay the EMT of the peritoneal membrane on dialysis (Li et al., 2017). And autophagy activation downregulated the production of oxygen free radicals, reducing intracellular oxidative stresses (Larabi et al., 2020). Taking together, our results indicated that autophagy was activated with the inhibition of the PI3K/AKT/mTOR signalling pathway, reducing intracellular oxidative stresses and preventing cellular EMT/PF damage caused by PD.

**FIGURE 7:**
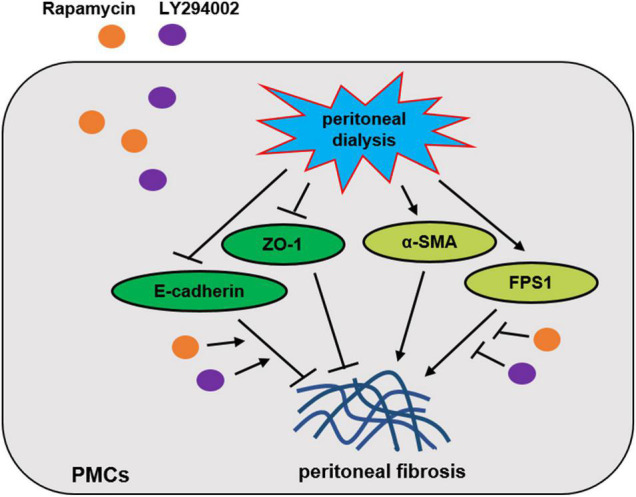
Proposed scheme for the mechanism by which Rapamycin and LY294002 obstructs peritoneal fibrosis *in vivo* and *in vitro*.

PI3K/AKT/mTOR is the classic signalling pathway regulating autophagy and induces the transdifferentiation of PMCs, which leads to PF. In conclusion, LY294002 and rapamycin promoted expression of autophagy-related proteins LC3-II/I, p62, and beclin-1 through regulating PI3K/AKT/mTOR signalling pathway to resist the process of peritoneal fibrosis *in vitro* and *in vivo* (Figure 7). Our results in further proved that intervening in this signalling pathway may become a research target for the prevention and treatment of PF. Due to the complexity of the molecular mechanism underlying PF development, more investigations are needed in future to explore additional evidences.

## Data Availability Statement

The datasets presented in this study can be found in online repositories. The names of the repository/repositories and accession number(s) can be found in the article/supplementary material.

## Ethics Statement

The animal study was reviewed and approved by The People’s Hospital of Suzhou New District.

## Author Contributions

MJ conceived and designed the study. HQ and LL analysed the data. SZ and DL conducted the experiments. MJ and DJ wrote the manuscript. All authors read and approved the final manuscript.

## Conflict of Interest

The authors declare that the research was conducted in the absence of any commercial or financial relationships that could be construed as a potential conflict of interest.

## Publisher’s Note

All claims expressed in this article are solely those of the authors and do not necessarily represent those of their affiliated organizations, or those of the publisher, the editors and the reviewers. Any product that may be evaluated in this article, or claim that may be made by its manufacturer, is not guaranteed or endorsed by the publisher.
